# Supplementary material for: An oral pH-responsive Streptococcus agalactiae vaccine formulation provides protective immunity to pathogen challenge in tilapia: A proof-of-concept study
Source: PLoS One. 2023 Mar 3;18(3):e0278277. doi: 10.1371/journal.pone.0278277 (PMC9983853; doi:10.1371/journal.pone.0278277)
Supplement: Attachment — Submitted filename: PONE-D-22-20929.docx [file pone.0278277.s001.docx]

**An oral pH-responsive *Streptococcus agalactiae* vaccine formulation provides protective immunity to pathogen challenge in tilapia: a proof-of-concept study**

Short title: An Oral pH-responsive vaccine for tilapia

Shazia Bashir^1¶^, Nguyen Ngoc Phuoc^2¶^, Tharangani Herath^3^, Abdul Basit^1^, Ruth N Zadoks^4^*, Sudaxshina Murdan^1^*

^1^UCL School of Pharmacy, 29-39 Brunswick Square, London, UK

^2^Faculty of Fishery, Hue University of Agriculture and Forestry, Hue University, Hue, Vietnam

^3^Department of Animal Health, Behavior and Welfare, Harper Adams University, Newport, Shropshire, UK

^4^Sydney School of Veterinary Science, Faculty of Science, The University of Sydney, Camden, NSW, Australia

^¶^First co-authors: these authors contributed equally to this work

*Corresponding authors

Email: [Ruth.zadoks@sydney.edu.au](mailto:Ruth.zadoks@sydney.edu.au) (RNZ)

Email: [s.murdan@ucl.ac.uk](mailto:s.murdan@ucl.ac.uk) (SM)

# Abstract

Intensive tilapia farming has contributed significantly to food security as well as to the emergence of novel pathogens. This includes *Streptococcus agalactiae* or Group B *Streptococcus* (GBS) sequence type (ST) 283, which caused the first known outbreak of foodborne GBS illness in humans. An oral, easy-to-administer fish vaccine is needed to reduce fish mortalities, economic loss, antimicrobial use, and the risk of zoonotic transmission associated with GBS. Our aim was to develop an oral vaccine formulation, which would only release its vaccine cargo at the site of action i.e., in the fish gastro-intestinal tract (GIT), rather than in the surrounding water, to reduce vaccine wastage in the aquatic environment. The study was designed as a proof-of-concept to evaluate whether the outcomes of the investigation provided sufficiently encouraging data to develop a follow-on study. The vaccine, formalin-inactivated *S. agalactiae* ST283, was entrapped within microparticles of Eudragit® E100, a polymer which is soluble at and below pH 5, e.g., in the fish’s stomach, but insoluble at the pH of the surrounding water. The double-emulsification solvent evaporation method was used to encapsulate the vaccine into microparticles. Upon exposure to an acidic medium (simulating the environment in tilapia stomach where pH was found to be between 1.5 and 3.9), the size of the vaccine-loaded microparticles decreased rapidly, reflecting microparticle erosion and release of the vaccine cargo. *In vivo* studies in tilapia showed that oral administration of vaccine-loaded microparticles to fish provided significant protection from homologous pathogen challenge by immersion compared to the control groups which received blank microparticles or buffer, reducing mortality from 70% to 20%. The high efficacy shows the promise of the vaccine platform developed herein, which might be adapted for other bacterial pathogens and other fish species.

**Keywords**: vaccine, tilapia, fish, streptococcosis, microparticles, Eudragit® E100, oral
1. Introduction

Aquaculture is a crucial part of the economy of low and-middle income countries (LMICs), including Vietnam, employing and feeding millions of people (1). Tilapia (*Oreochromis* spp.) – is eminently suitable for aquaculture due to its fast growth, ability to survive in poor water conditions, to breed throughout the year, to produce high numbers of eggs, and to eat a wide range of feeds (2), and is the third most common fish species in aquaculture (3) with global production volumes reaching 4.5 million tonnes in 2018. In addition to producing more food, intensification of tilapia farming also increases the risk of infectious disease outbreaks, causing significant waste and economic loss. Streptococcosis, the most important disease of tilapia (4), is primarily caused by *Streptococcus agalactiae*, known in human medicine as group B *Streptococcus* (GBS). Streptococcosis mostly occurs when fish are stressed due to, for example, an increase in water temperature, sub-optimal oxygen levels or overcrowding and can lead to high levels of disease and mortality, especially towards the end of the production cycle, aggravating the losses incurred (4, 5). In 2015, GBS sequence type (ST)283 caused an outbreak of invasive disease in people in Singapore. Uniquely, those affected had few underlying comorbidities, and the route of exposure was foodborne, traced back to the consumption of raw fish with GBS (5). Based on evolutionary analysis of genomic data, GBS sequence type (ST) 283 emerged around the time of intensification of aquaculture in the early 1980s and is now a recognized cause of severe invasive disease in both fish and humans in Southeast Asia (6, 7). Its unusual – foodborne - route of transmission has led the Food and Agricultural Organisation (FAO) of the United Nations to publish a Risk Profile on its characteristics and role as a human health hazard in 2021 (8).

Antimicrobials are commonly used, especially in LMICs to control infections in fish (1, 9). This is undesirable, both from fish, and public, health perspectives, as infected fish have diminished appetite and therefore do not benefit from oral antimicrobial treatment, whilst the release of antimicrobials in the aquatic environment may exert selection for antimicrobial-resistant pathogens (1, 10, 11). Prophylactic vaccination is a far more desirable alternative (12), and many types of fish vaccines for delivery by injection, immersion, orally, or as spray have been developed (13). Most vaccines that are available for tilapia are injectable formulations (14, 15), and thus are only suitable for fish that are large enough to be handled and anaesthetized. In addition, the injection of vaccines into each fish demands individual handling of fish, which can lead to handling-related stress, immunosuppression and mortalities (14), and is labour-intensive, making it cost-prohibitive for low value fish such as tilapia.

Oral vaccination is preferable, and is perhaps, the most suitable method for mass immunization of any fish species and has been proven to be effective in protecting against mass mortality, making it an ideal route for delivering vaccine antigens (16). In addition, oral administration stimulates mucosal immunity, i.e., the appearance of antigen specific antibodies in skin mucus, bile, or intestine in several fish species (17, 18). The presence of a mucosal immune system makes oral vaccination highly relevant because the first contact with the pathogens generally occurs through mucosal surfaces (19, 20).

Several strategies have been evaluated to enhance the efficacy of oral vaccines, for example, by inhibiting antigen degradation in the fish stomach via the concomitant administration of antacids and antiproteases, use of ‘prills’ (pellets comprising lyophilized vaccine incorporated into a matrix of saturated long chain fatty acids), coating vaccine granules and pellets with acid-resistant polymers, use of adjuvants and encapsulation of vaccine antigens into vaccine carriers such as microparticles, beads and liposomes (21-31). Targeted mucosal vaccine delivery can be achieved by exploiting the difference in the pH of the aquatic environment (pH 7-8) and that of the fish stomach contents (acidic pH) and entrapping the vaccine within pH-responsive carriers such as microparticles. Targeted delivery to specific parts of the GIT has been demonstrated in human medicine (32), and it is expected that the approach is transferable to aquaculture. Indeed, various studies have developed intestine-targeted fish vaccines, using pH sensitive polymers that are only soluble above pH 5, to fabricate vaccine carriers, which are loaded into fish feed pellets (33). The vaccine-loaded food pellets are fed to the fish by way of dispersion in the fish tanks during regular feeding times. This approach has the advantage of ensuring that the vaccine delivery vehicle bypasses the fish stomach to avoid exposure of the antigen to acid and enzymatic degradation and will only dissolve in the fish intestines. However, polymers soluble in the intestinal environment of the fish (~ pH 6.9-7.9, see Fig 6) will also be soluble in water, thus vaccines fabricated from these types of polymers will also be subjected to potential dissolution in fish water tanks causing leaching from feed and loss of antigen. Consequently, to induce appropriate levels of immune protection, the tilapia must be fed with vaccine-loaded feed for multiple consecutive days. For example, Halimi *et al.* (34) developed vaccine-loaded fish feed pellets coated with Eudragit L30D-55 for rainbow trout. The relative percentage survival (RPS) was found to be very high for the vaccinated groups (85 ± 7% for *S. iniae*, 72 ± 8% for *Lactococcus garviae*) in comparison to the unvaccinated group, but this was only achieved with daily immunization for 14 consecutive days. Furthermore, the possibility of the polymer-coating dissolving in water was not addressed. Hayat *et al.* (35) assessed the effectiveness of a formalin-killed whole cell *S. iniae* vaccine loaded within food pellets, on red hybrid tilapia. Several immunization regimens were tested and it was demonstrated that the lowest mortality was achieved for the fish group that were orally vaccinated by feeding for 9 consecutive days and then received oral boosters (by gavage) on days 14 and 21. These studies evaluating the efficacy of feed-based vaccines demonstrate successful immunization strategies but the resultant experimental outcomes reveal that protection and immunity is difficult to achieve when applying the conventional, and more cost-effective, vaccine strategy of an initial single dose as prime followed by one booster dose.

The aim of the work described herein was to develop and demonstrate proof-of-concept of a vaccine formulation that, if loaded into fish feed pellets, will not dissolve in the aquatic environment (river water/fish tanks etc.), and will only dissolve in the fish stomach, thereby preventing vaccine loss into the environment and enabling lower antigen doses and fewer antigen administrations to be used. Eudragit E100, a pH-sensitive cationic polymer based on dimethylaminoethyl methacrylate, butylmethacrylate and methylmethacrylate, which only dissolves in acidic environments (at and below pH 5 (36)) and is generally recognized as safe (GRAS) (37), was selected as the pH-responsive polymer to encapsulate the vaccine antigen. While Eudragit-based pH-responsive films, around vaccine granules and beads and around vaccine-coated feed pellets, have been investigated as a means of protecting oral vaccines from degradation in fish stomach (34, 38, 39) to our knowledge, Eudragit E100 has not been used to form the matrix of particulate oral vaccine formulations in aquaculture. If an appropriate polymer is selected, vaccine microencapsulation can confer a number of advantages, such as protection against loss of antigen in the aquatic environment for feed-based vaccines, shielding from degradation and clearance in the fish stomach and intestines, the possibility of co-encapsulation of antigens and immune modulators, targeting to, and enhanced uptake by antigen presenting cells and enhancement of vaccine immunogenicity (40).

Our aim was achieved. In this paper, we report Eudragit E100 microparticle preparation and characterization, measurements of pH in the tilapia gastro-intestinal tract (GIT) and fish tanks, and evaluation of vaccine efficacy of the pH-responsive vaccine-loaded microparticles using an experimental challenge model. Tilapia were used as model fish because of their tractability in vaccine studies and their economic importance (3, 41); whole cell inactivated GBS was used as the model vaccine, because bacterin vaccines have been proven to be efficacious in tilapia (41); and the GBS ST283 strain was used because of its significance to fish and human health in Southeast Asia, a major player in aquaculture/tilapia production .

# 2. Materials and Methods

## 2.1 Materials

Eudragit E100 was kindly gifted by Evonik (Essen, Germany). Other chemicals were purchased as follows: Polyvinyl alcohol (PVA, BDH Laboratory supplies, Poole, England), Sorbitan monostearate (Thermo Fisher Scientific, Heysham, UK), Absolute Ethanol (≥99.8%, Sigma-Aldrich, UK), and Dichloromethane (Sigma-Aldrich, UK). All other materials used were of laboratory grade quality.

### 2.1 Group B Streptococcus isolates used for vaccination and for challenge:

The vaccine strain, GBS isolate MRI Z2-388, belongs to ST283, and was originally isolated by the authors from the brain of a clinically affected tilapia in the Mekong Delta area, Vietnam, in 2016. To preserve the structural integrity of the bacteria, they were formalin-inactivated in 2% paraformaldehyde (1 h at room temperature, then 24 h at 4^o^C), followed by suspension at 10^9^ CFU/mL in 0.1 M PBS, pH 7.4.

The challenge bacteria, GBS isolate 0101030, also belongs to the ST283 strain, and was also isolated by the authors from the brain of a clinically affected tilapia in the Mekong Delta area, Vietnam (Add reference or NCBI GenBank accession number). Both isolates were identified to species and strain level by colony morphology, Gram stain, and multi-locus sequence typing (MLST) as described in (6).

### 2.2 Characterisation of GBS bacteria for particle size and surface charge

To determine the morphological characteristics of the formalin-inactivated GBS organisms used in the vaccine formulation, samples of a dilute dispersion of GBS (in de-ionized water, 10^7^ CFU/ml) were mounted onto aluminum stubs and allowed to dry overnight before being sputter coated with gold in a high-vacuum evaporator for 3 min at 30mA (Emitech K550, Ashford, England) and photographed using a scanning electron microscope (SEM, Philips XL30, Eindhoven, Holland).

The particle size and zeta potential of GBS organisms was measured by dynamic light scattering using the Zeta sizer Ultra (Malvern instruments Ltd, Worcestershire, UK), using a GBS suspension (0.7mL at 10^6^ CFU/mL in deionized water).

### 2.3 Preparation of GBS-loaded, and of blank Eudragit E100 microparticles

Both blank and GBS-loaded microparticles were produced using the emulsification solvent evaporation (ESE) technique. Blank microparticles were produced using the single ESE method, while GBS-loaded microparticles were prepared using the double ESE technique as follows.

*GBS-loaded microparticles* were prepared using a water-in-oil-in-water (w/o/w) double-emulsion solvent evaporation technique adapted from (42). The primary emulsion was formed by adding 300 µL of GBS suspension (10^9^ CFU/mL) dropwise (slowly over a 30min period) to an organic solution (3mL) of Eudragit E100 (7.6% w/v) and sorbitan monostearate (2.5% w/v) dissolved in a dichloromethane-ethanol (1:1 v/v) mixture under continuous stirring at 800 rpm for approximately 30 min. The resultant primary emulsion was homogenized for 2 min (6000rpm, IKA T10 basic Ultra-Turrax), then added dropwise (over a period of approximately 30 min), to an aqueous 4% w/v PVA solution (6mL) under vigorous stirring at 800rpm. The emulsion was then left to stir overnight (at the same stirring speed), for the solvent to evaporate and the microparticles to harden. The resultant microparticles were washed and rinsed twice (with 0.1 M PBS, pH 7.4) by centrifuging at 10,000 g for 10 min (Sigma Laborzentrifugen GmbH, Germany). After every wash cycle, the supernatant was discarded to remove excess PVA and replaced with fresh 0.1M PBS. The microparticle pellet was collected and lyophilized for 48 h at -85^o^C (Alpha 1-4 LD plus, Christ, Germany), then stored at room temperature until further use.

*Blank Eudragit E100 microparticles* were prepared by slowly adding 3 mL of a solution of Eudragit E100 (7.6 %w/v) in dichloromethane-ethanol (1:1 v/v) dropwise (over 30 min) to an aqueous PVA (6mL, 4% w/v) solution at 800 rpm. The mixture was left to stir overnight for solvent evaporation to occur and for microparticles to harden. The resultant microparticles were washed, lyophilized, and stored, as described for GBS-loaded microparticles.

### 2.4 Characterisation of GBS-loaded, and of blank Eudragit E100 microparticles for size, surface charge, morphology and topography and pH-responsiveness

A mass of lyophilized microparticles (~0.1mg) was added to 10mL of 0.1M PBS and the mixture was manually agitated to obtain a homogenous dispersion that appears sightly opaque. A sample of the resultant suspension was characterized for size and surface charge using the Zeta sizer Ultra (Malvern instruments Ltd, Worcestershire, UK).

Eudragit E100 microparticle morphology and surface topography was examined by scanning electron microscopy (SEM). A drop of a dilute microparticle dispersion (in deionized water) was examined as described above in Section 2.2.

The pH-responsiveness of Eudragit E100 microparticles was evaluated by placing the particles in an acidic medium (to simulate the conditions of the fasted fish stomach) and monitoring the changes in particle size with time, using the Zeta sizer Ultra. Briefly, 500µL of a microparticle dispersion (2mg in 10mL of de-ionised water) was added to 0.1 M hydrochloric acid (10mL, pH 1.3) under constant stirring at 300rpm. At time intervals, aliquots (0.7mL) were withdrawn from the mixture and the particle size was measured using the Zeta sizer Ultra. Removed aliquots were replaced with an equivalent volume of 0.1 M HCl to maintain sink conditions.

### 2.5 Experiments in tilapia.

### 2.5.1 Fish and rearing conditions

Red tilapia (*Oreochromis* sp.) was purchased and transported from the Provincial Breeding Centre of Thua Thien Hue at Cu Chanh Commune, Thua Thien Hue province, Vietnam. The fish were starved for 24 h prior to transportation and maintained at 15 °C during the 30-minute journey to the Laboratory of Fish Pathology, Hue University of Agriculture and Forestry (HUAF), Hue City, Vietnam. Fish were housed in 1000 L fiber-glass tanks using continuous flow-through water at a flow rate of 0.38 L min^-1^ at 28 °C ± 2 °C, and fed with commercial tilapia diet (Aquaxcel 7444, Cargill, Vietnam) at 2% of body weight for 14 days prior to commencing experiments. Mean body weight of the tilapia used in this study was 15 ± 2 g on the day of the vaccination. Before vaccination, the population was tested for the absence of *S. agalactiae* based on clinical monitoring by euthanasia of 5 fish, followed by directly streaking the kidney and the brain onto tryptone soya agar (TSA, Himedia) as described previously in (6). All experiments were conducted with approval of the newly established Animal Ethics Committee at HUAF, and in accordance with UK Home Office standards.

### 2.5.2 Measurement of the pH of tilapia gastrointestinal tract (GIT) contents in the fasted and fed states

The pH of the GIT contents of tilapia was measured using a HI 2210-02 portable pH meter (Hanna, Taiwan) and a 3-mm diameter pH probe (H1095B microelectrode, Hanna Instruments, Scientific Laboratory Supplier, UK). Tilapia (5 fasted, i.e. before feeding, and 5 fed, i.e., 1.5 h after feeding) were euthanized by exposure to an overdose of Aqui-S in an immersion bath (Bayer, Vietnam) at a concentration of 150 mg L^-1^ for 60 minutes as per (43). Within the next 5–10 min, the pH of the oral cavity (anterior and posterior mouth) was measured (Fig. 1A). Subsequently, the carcasses were carefully dissected, the GIT was removed and cut into sections to enable measurement of pH at multiple points along its length, namely anterior stomach, posterior stomach, and at four points in the intestine (shown in Fig. 1B). The pH of each section was measured in each of the 5 fish, by inserting the pH probe into the GIT contents.

***Figure 1: pH measurement of gastrointestinal tract of tilapia: A – Mouth; B – 1. Anterior stomach, 2. Posterior stomach; 3,4,5,6: intestine.***

### 2.5.3 Vaccination and challenge studies

*Fish vaccination and challenge experimental design*

To evaluate the efficacy of the oral GBS vaccine formulation prepared in Section 2.3, a total of 300 red tilapia were divided into three treatment groups consisting of 100 fish per group. The three treatment groups were: (1) Negative control, i.e., fish received mock vaccination with PBS; (2) Delivery system control, i.e., fish received 0.2 mL of a suspension of blank Eudragit microparticles; (3) Vaccinated, i.e., fish received 0.2 mL of a suspension of GBS-loaded Eudragit microparticles. Each treatment group of 100 fish was held in four experimental tanks (i.e. 25 fish/tank and 4 replicates per treatment). The number of fish per treatment group was calculated based on the study of Shoemaker *et al*. (44) and using (45). In addition to using the two negative control groups, namely fish dosed with PBS only and fish dosed with blank Eudragit microparticles, we also considered a third control, namely un-encapsulated GBS. However, we chose not to include the latter group, in line with the 3R principles, as our aim was **not** to see whether GBS encapsulation enhances vaccine efficacy, but rather to test the potential of encapsulated GBS to generate protective efficacy and avoid being released in the aquatic environment.

Experimentation on fish were conducted in the fasted state i.e., one hour prior to the first meal of the day. Before oral administration of the GBS vaccine formulation, the fish were anaesthetized with Aqui-S^TM^ at a dose of 0.02 mL in an immersion bath for 10 minutes. Fish in treatment groups 1, 2 and 3 were then orally dosed (0.2mL, by gavage) with PBS and blank and GBS-loaded microparticles (dose of GBS was 5 x 10^6^ CFU) respectively, using a 1 mL syringe without a needle. All the fish were placed into flow-through experimental tanks (120 L) and observed twice daily for 21 days.

Prior to performing the challenge study, *S. agalactiae* was passed through naïve tilapia twice to restore pathogen virulence after storage, as previously described in (6). On Day 21 post vaccination, fish in tanks were challenged by immersion. They were transferred to 10 L tanks containing GBS bacteria at 3.5 × 10^6^ CFU mL^-1^, removed after 30 minutes and placed back into the original flow-through experimental tanks and observed for a further 21 days. The bacterial concentration used for challenge was determined from pilot studies and was designed to give a 70% mortality in the control groups (6).

Throughout the experiment, the fish were fed a commercial tilapia diet (Aquaxcel 7444, Cargill, Vietnam) at 2% of body weight and examined twice daily for gross clinical signs of disease. If moribund or freshly dead fish were found in the tanks, they were killed using a Schedule 1 procedure (46). On Day 42 (i.e. 21 days after challenge), the surviving fish were euthanized using the same Schedule 1 method. Bacterial recovery was attempted from moribund fish and from 3 surviving fish per tank by culturing the head kidney on trypticase soy agar (6). Relative percent of survival (RPS) was calculated according to the formula proposed by Amend (1981) (47).

$$RPS=1-(\frac{{cum.mort}_{vaccinated}}{{cum. mort}_{control}} )$$

### 2.6 Statistical analyses

Data analysis was conducted using Excel and SPSS v 26. Repeated measures ANOVA, followed by post-hoc Tukey was conducted to establish significant differences, if any, in the pH of the gastro-intestinal tract in the fed and fasted states. One way ANOVA, followed by Duncan Test was conducted to compare fish mortality in the different groups upon challenge. Alpha level was set at 0.05 to define statistical significance.

# 3. Results

## 3.1 Morphology, size, and charge of GBS vaccine and of Eudragit E100 particles

Light and scanning electron microscopy of formalin-inactivated GBS bacteria used as the vaccine showed the characteristic cocci and pair or chain forming features, the chains being much reduced in length probably due to formalin exposure and other processing procedures (Fig. 2). The surface of GBS was negatively charged (Table 1), which is characteristic of bacteria suspended in aqueous media where the various functional groups present on the external cell wall surface will ionize depending on the nature of the external environment (48) (49).

***Figure 2: Light (A) and scanning electron (Bi-ii) micrographs of the formalin-inactivated bacterial used in vaccine formulations. Scale bar indicates 100 µm (A); 5 µm (B)***

***Table 1: Particle size measured by dynamic light scattering (mean ± SD), n= 12***

|  | **Mean particle size (µm) (±SD)** | **Zeta potential (mV)** |
| --- | --- | --- |
| **GBS (formalin inactivated) bacteria** | 2.46 (±0.13) | -23.42 (±1.22) |
| **Blank Eudragit E100 microparticles** | 2.90 (±0.91) | 41.77 (±4.25) |
| **GBS-loaded Eudragit E100 microparticles** | 8.92 (±2.94) | -70.39 (±3.86) |

Blank Eudragit E100 microparticles (Fig. 3) were spherical in shape with a rough surface, a mean particle size of approx. 3 µm and a surface charge of 42 mV (Table 1). In contrast to the blank microparticles, GBS-loaded microparticles were distinctly different, with a much larger particle size and a negative surface charge (Table 1). In addition, while the blank Eudragit E100 particle surface was smooth and spherical, in comparison the GBS-loaded particles had irregular shapes with indentations which gave the particles the appearance of deflated balls (Fig 4).

***Fig 3: Scanning electron micrograph of blank Eudragit E100 microparticles. Scale bare indicates 5 µm.***

***Fig 4: Scanning electron micrograph of GBS-loaded Eudragit E100 microparticles after freeze-drying. Scale bare indicates 10 µm***

## 3.2 pH-responsiveness of E 100 microparticles

Upon exposure to 0.1M HCl (simulating the acidic environment in the fasted tilapia stomach), the particle size of both blank and GBS-loaded microparticles decreased within 10 minutes, (Fig. 5), indicating rapid dissolution of the polymeric microparticles. Particle size measurements gave readings approaching zero for the blank Eudragit E100 microparticles, indicating almost complete dissolution of the polymer within 10 minutes. In contrast, acidic exposure of the GBS-loaded microparticles resulted in reduction in size until a plateau of around 2 µm was reached. Particles of 2 µm are most likely to be the previously-encapsulated bacteria which have been released from Eudragit E100 microparticles upon exposure to acidic conditions. Figure 5 thus demonstrates the ability of Eudragit E100 microparticles to release their entrapped cargo in the presence of an acidic environment.

***Fig 5: Change in particle size upon exposure to 0.1M HCl, which is simulating the acidic environment in a fasted tilapia stomach. GBS-loaded Eudragit E100 microparticles (*o*) ; Blank Eudragit E100 microparticles (▪); Means are shown. Error bars reflect the SD. N=12***

## 3.3 pH of tilapia’s stomach and intestine

The pH of the contents in the different tilapia GIT sections are shown in Figure 6. In both the fasted and fed states, the pH of the GIT contents changed significantly along the GIT tract (repeated measures ANOVA, p<0.05), from neutral in the mouth, to acidic in the stomach, rising back to neutral and slightly alkaline in the mid-intestine and falling slightly below neutral at the anus. Post-hoc analysis showed that in both fed and fasted states, the stomach pH was significantly lower than the pH in the other sections of the GIT (p<0.05). When the whole pH profile along the GIT is considered, mixed between-within subjects analysis of variance showed that the profiles in the fed and fasted states were significantly different from each other (p<0.05). Furthermore, the clear difference in pH range along the GIT of tilapia observed in Fig. 6 demonstrates the usefulness of selecting a pH-sensitive polymer such as Eudragit E100 for targeted delivery to the tilapia stomach. Since it is only soluble below pH 5, this polymer is likely to remain insoluble in water, as well as in the fish mouth.

***Fig 6. pH in the different sections of the GIT of fasted and fed tilapia (mean ± SD), N=5***

## 3.4 Cumulative percentage mortality in vaccination and challenge experiments

The challenge study was successful, as demonstrated by 70.7 ± 4.2 % mortality in the control group (Fig. 7). The group which received the blank Eudragit E microparticles had similarly high mortality (67.9 ± 4.6 %) (ANOVA, Duncan Test, p>0.05), indicating that blank microparticles do not induce protection against *S. agalactiae* challenge. In contrast, mortality in the vaccinated group was only 22.6 ± 4.6% (ANOVA, Duncan Test, p<0.05), equating to RPS = 0.70, and indicating highly successful protection against homologous challenge. The difference among the groups was also noticeable in the onset of mortality (day 2 post challenge (p.c.) for negative and blank Eudragit E100 microparticle control groups vs day 4 p.c. for the vaccinated group) and in the last observed mortality (day 14 for negative and Eudragit E100 microparticle control groups vs day 12 for vaccinated group).

***Fig. 7 Cumulative percentage mortality in the 3 groups following oral gavage with: (ai) PBS in the negative control group; (aii) Blank Eudragit E100 microparticles; (b) GBS-loaded Eudragit E100 microparticles, and challenge by immersion using a homologous strain of S. agalatiae sequence type on day 21 post-gavage. Error bars represent SD from 4 replicate tanks (25 fish per tank) for each of 3 treatment groups.***

The clinical signs commonly associated with *S. agalactiae* infection were observed after experimental challenge in each treatment group (Fig. 8), including unilateral or bilateral opacification of the eye and eye haemorrhage (Fig. 8). Pure cultures of bacteria identified as *S. agalactiae* were recovered from 100% of moribund and fresh dead fish. No clinical signs of disease or bacteria were observed or recovered from any of the survivors.

***Figure 8: Bilateral exophthalmia of the eye (pop-eyes) and eye hemorrhages (left eye) were found in moribund fish with S. agalactiae infection.***

# 4. Discussion

We report, for the first time, the use of Eudragit E100 polymer to form the matrix of GBS-loaded microparticles via a simple one-step preparation, for oral vaccination in aquaculture. So far, in experimental fish oral vaccines, alginate is the most commonly used polymer to encapsulate antigens, although chitosan and poly D,L-lactide-co-glycolic acid (PLGA) have also been used (22) (25) (26-29) (30, 31). This work shows that Eudragit E100 is a promising polymer for formulating inactivated GBS into a vaccine formulation.

GBS-loaded Eudragit E100 microparticles were successfully prepared using the double-emulsion solvent evaporation technique: a GBS suspension in phosphate buffer was emulsified into a water-in-oil (w/o) emulsion, which was itself emulsified into a water-in-oil-in-water (w/o/w) emulsion, which was left to stir overnight for solvent evaporation to take place and for the polymer to precipitate into microparticles entrapping GBS. In contrast, blank Eudragit E100 microparticles were prepared using a single-emulsion solvent evaporation as there was no need for the internal aqueous phase: an organic solution of the polymer was emulsified into an oil-in-water (o/w) emulsion, which was left to stir overnight for solvent evaporation to take place and for the polymer to precipitate into microparticles. The absence of the internal aqueous phase during blank microparticle preparation had a significant influence on particle properties, such that blank microparticles were much smaller than GBS-loaded microparticles (2.9µm vs 8.9µm) and had a positively charged surface (zeta potential = 42 mV) reflecting the cationic nature of Eudragit E100 (a copolymer based on dimethylaminoethyl methacrylate, butyl methacrylate, and methyl methacrylate) conferred by ionization of the amine groups in the repeating units of the polymer backbone.

In contrast to blank microparticles, GBS-loaded microparticles had a negative charge (zeta potential being -70 mV). This is most likely to be due to movement of the phosphate ions from the internal aqueous phase (GBS in phosphate buffer) to the particle surface, as the inner aqueous phase is partly expulsed during solvent elimination and shrinkage and polymer precipitation (50). Particle morphology was also affected, blank particles being spherical (Fig. 3) while the GBS-loaded particles had irregular shapes (Fig. 4). The difference in particle morphology is directly related to whether particles were formed by single or double ESE method, single ESE being known to result mostly in spherical particles, while double ESE results in mixtures of honeycomb, deflated ball and plain structures, depending on the inner aqueous phase content of the emulsion droplet and on the size of the inner microdroplet relative to the emulsion droplet (50).

Both blank and GBS-loaded Eudragit E100 microparticles were pH-responsive, dissolving in acidic media (Fig. 5), as intended. The rapid decrease in microparticle size within 10 minutes of acidic exposure indicates that the vast majority of the Eudragit E100 polymer had dissolved within the first 10 minutes. The acidic pH in the tilapia stomach and neutral-to-weakly alkaline pH in the intestine measured in the current study (Fig. 6) reflecting similar measurements in other species of tilapia, such as *Tilapia guineensis* and *Sarotherodon melanothron* and other fish species, such as the *Liza falcipinnis* grey mullet (51), suggests that the particles should dissolve quickly in the stomach after administration via oral gavage.

According to the literature, gastric emptying time in tilapia (fasted for 12 h, then fed a commercial diet) ranges from 4 to 15 h, with a mean of 8 h (52), with a high degree of evacuation from the stomach within 5 h and complete emptying after 16 h (53). Such long gastric residence time indicates that Eudragit E100 microparticles will have sufficient time to dissolve in the acidic milieu, and for GBS-loaded Eudragit E100 microparticles to release the entrapped vaccine cargo. Given the higher acidity of the fasted stomach (Fig. 6), in the vaccination experiment, Eudragit E100 microparticles were orally administered one hour prior to feeding, to further ensure rapid release of the vaccine from the GBS-loaded Eudragit E100 microparticles in the fish stomach.

While oral administration of GBS-loaded Eudragit E100 microparticles was effective at reducing mortality upon pathogen challenge, oral administration of blank microparticles resulted in similar mortality compared to fish which only received the buffer (control group, Fig. 7). This shows that microparticles by themselves were not responsible for any protection. Although, microparticulate systems are known for their immunostimulatory properties which further adds to the benefit of delivering oral vaccines using this technique (54), the results obtained in this study indicate that the blank microparticles provided no significant immuno-protection and will have most likely dissolved almost instantly in the gastric fluid.

For the GBS-loaded Eudragit E100 microparticles, which reduced the mortality rate upon pathogen challenge (Fig. 7), we calculate a vaccine efficacy of 70%, efficacy being calculated as % reduction in mortality compared to the control unvaccinated group. Embregts and Forlenza (33), reviewed oral GBS vaccination studies in tilapia and report 25% to 85% protection, whereby the higher values were generally achieved with multiple vaccine doses administered, and only one vaccine regime in one study achieved 100% protection (33). Protection of 70% or more, generally required more vaccine administrations or a higher vaccine dose (CFU/fish) than those used in our protocol. Moreover, an RPS of 70% after a single oral vaccine administration of only 10^6^ CFU/fish suggests that the pH-sensitive particles may have considerable benefit compared to other oral delivery systems.

Teleost fish have a morphologically and functionally different gut associated lymphoid system (GALT) than mammals, with a distinct lack of a concentrated lymphoid tissue mass such as Peyer’s patches found in humans. Instead, lymphoid tissue in the fish gut is diffuse throughout the GIT, consisting mainly of isolated lymphoid patches. In general, development of efficacious oral vaccine is considered challenging due to antigen breakdown in the harsh gastric environment and the potential of antigenic tolerance developing in the gut (33), as well as a general lack of understanding of how tilapia immune system functions (55, 56). Our study based on encapsulation technology sheds some light on how to overcome some of the challenges and bottlenecks of oral vaccine development for fish. We have shown that protective immunity is possible when formalin-inactivated GBS is liberated from the Eudragit E100 polymeric carrier and released into the acidic environment of the fish stomach. To develop the findings from this investigation, further studies must be conducted to evaluate the efficacy of the encapsulated GBS when incorporated in fish feed and compare to unencapsulated GBS. Feed-based vaccine is the most practical, economical and welfare friendly method of vaccinating the tilapia fish. Other avenues for potential refinement of the vaccination protocol would be the inclusion of an adjuvant to stimulate mucosal immunity and simplification of the GBS-inactivation protocol. If heat-killed GBS could be used rather than formalin-fixed GBS, this would simplify vaccine production and reduce the cost of goods, which is an important consideration for production of economically viable vaccines. In addition, the efficacy of multivalent vaccines should be explored, considering that there are three major lineages of GBS that can cause streptococcosis in tilapia (57, 58). Commercial vaccines are currently mostly sold as providing protection against biotype 1 (non-haemolytic; equivalent to serotype Ib, clonal complex (CC) 552 (57)) or biotype 2 (haemolytic; serotype Ia, CC7 and serotype III, CC283 (57)) with limited cross-protection between biotypes. In the current study, we evaluated protection against homologous challenge (ST283 used as vaccine strain and as challenge strain). Further work is needed to evaluate whether mono- or multi-valent vaccines administered orally using pH-responsive particles could provide protection against heterologous challenge or multiple strains. Finally, our challenge model was based on immersion, which is a more natural route of exposure than intraperitoneal injection, but not as natural as cohabitation with infected fish. Thus, evaluation of vaccine efficacy in a cohabitation challenge model could be considered as an intermediate step between evaluation based on immersion exposure and field studies.

# 5. Conclusions

In this manuscript, we describe the development, and show proof-of-concept of an oral vaccine for the prevention of streptococcosis in tilapia. The vaccine formulation, composed of pH-responsive microparticles entrapping killed *S. agalactiae,* provided very good immune protection against homologous challenge in tilapia. Further vaccine refinements, notably in-feed administration, and evaluation of costs and benefits of the pH-responsive particles are logical next steps in development of this concept, which may also have applicability across other pathogen- and host-species.

# References

1.	Reverter M, Sarter S, Caruso D, Avarre J-C, Combe M, Pepey E, et al. Aquaculture at the crossroads of global warming and antimicrobial resistance. Nature Communications. 2020;11(1):1870.

2.	Nandlal S, Pickering T. Tilapia Fish Farming in Pacific Island Countries Vol. 1: Tilapia Hatchery Operation2004.

3.	The state of world fisheries and aquaculture. Food and Agriculture Organisation of the United Nations; 2020.

4.	Maulu S, Hasimuna OJ, Mphande J, Munang'andu HM. Prevention and Control of Streptococcosis in Tilapia Culture: A Systematic Review. Journal of Aquatic Animal Health. 2021;33(3):162-77.

5.	MNA Amal MZ-S. Streptococcosis in Tilapia (Oreochromis niloticus) (italic): A Review. Pertanika J Trop Agric Sci. 2011;34(2):195-206.

6.	Phuoc NN, Linh NTH, Crestani C, Zadoks RN. Effect of strain and enviromental conditions on the virulence of Streptococcus agalactiae (italic) (Group B Streptococcus; GBS) in red tilapia (Oreochromis sp.). Aquaculture. 2021;534:736256.

7.	Barkham T, Zadoks RN, Azmai MNA, Baker S, Bich VTN, Chalker V, et al. One hypervirulent clone, sequence type 283, accounts for a large proportion of invasive Streptococcus agalactiae (italic) isolated from humans and diseased tilapia in Southeast Asia. Plos Neglected Tropical Diseases. 2019;13(6).

8.	Nations FaAOFotU. Risk profile - Group B Streptococcus (GBS) - Streptococcus agalactiae (italic) sequence type (ST) 283 in freshwater fish. Bangkok; 2021.

9.	Ayukekbong JA, Ntemgwa M, Atabe AN. The threat of antimicrobial resistance in developing countries: causes and control strategies. Antimicrobial Resistance & Infection Control. 2017;6(1):47.

10.	Harikrishnan R, Balasundaram C, Heo M-S. Fish health aspects in grouper aquaculture. Aquaculture. 2011;320(1):1-21.

11.	Xiong W, Sun Y, Zhang T, Ding X, Li Y, Wang M, et al. Antibiotics, Antibiotic Resistance Genes, and Bacterial Community Composition in Fresh Water Aquaculture Environment in China. Microbial Ecology. 2015;70(2):425-32.

12.	AQUAVAC Strep *Sa*1: MSD; 2017 [Available from: <https://www.aquavac-vaccines.com/products/aquavac-strep-sa1/>.

13.	Lee PT, Yamamoto FY, Low CF, Loh JY, Chong CM. Gut Immune System and the Implications of Oral-Administered Immunoprophylaxis in Finfish Aquaculture. Frontiers in Immunology. 2021;12.

14.	Munang'andu HM, Paul J, Evensen O. An Overview of Vaccination Strategies and Antigen Delivery Systems for Streptococcus agalactiae (italic) Vaccines in Nile Tilapia (Oreochromis niloticus) (italic). Vaccines. 2016;4(4).

15.	Miccoli A, Manni M, Picchietti S, Scapigliati G. State-of-the-Art Vaccine Research for Aquaculture Use: The Case of Three Economically Relevant Fish Species. Vaccines. 2021;9(2) complete this reference.

16.	Assefa A, Abunna F. Maintenance of Fish Health in Aquaculture: Review of Epidemiological Approaches for Prevention and Control of Infectious Disease of Fish. Veterinary Medicine International. 2018;2018:5432497.

17.	Rombout JW, Blok LJ, Lamers CH, Egberts E. Immunization of carp (Cyprinus carpio) (italic) with a Vibrio anguillarum (italic) bacterin: indications for a common mucosal immune system. Dev Comp Immunol. 1986;10(3):341-51.

18.	Rombout JHWM, van den Berg AA, van den Berg CTGA, Witte P, Egberts E. Immunological importance of the second gut segment of carp. III. Systemic and/or mucosal immune responses after immunization with soluble or particulate antigen. Journal of Fish Biology. 1989;35(2):179-86.

19.	Joosten PHM, Tiemersma E, Threels A, Caumartin-Dhieux C, Rombout JHWM. Oral vaccination of fish againstVibrio anguillarumusing alginate microparticles. Fish & Shellfish Immunology. 1997;7(7):471-85.

20.	Manganaro M, Ogra PL, Ernst PB. Oral immunization: turning fantasy into reality. Int Arch Allergy Immunol. 1994;103(3):223-33.

21.	Mutoloki S, Munang’andu HM, Evensen Ø. Oral Vaccination of Fish – Antigen Preparations, Uptake, and Immune Induction. Frontiers in Immunology. 2015;6(519).

22.	Bogwald J, Dalmo RA. Protection of Teleost Fish against Infectious Diseases through Oral Administration of Vaccines: Update 2021. International Journal of Molecular Sciences. 2021;22(20).

23.	Rivas-Aravena A, Sandino AM, Spencer E. Nanoparticles and microparticles of polymers and polysaccharides to administer fish vaccines. Biol Res. 2013;46(4):407-19.

24.	Ellis AE. Recent development in oral vaccine delivery systems. Fish Pathology. 1995;30(4):293-300.

25.	Behera T, Swain P. Alginate–chitosan–PLGA composite microspheres induce both innate and adaptive immune response through parenteral immunization in fish. Fish & Shellfish Immunology. 2013;35(3):785-91.

26.	Kitiyodom S, Kaewmalun S, Nittayasut N, Suktham K, Surassmo S, Namdee K, et al. The potential of mucoadhesive polymer in enhancing efficacy of direct immersion vaccination against Flavobacterium columnare (italic) infection in tilapia. Fish Shellfish Immunol. 2019;86:635-40.

27.	Rodrigues AP, Hirsch D, Figueiredo HCP, Logato PVR, Moraes ÂM. Production and characterisation of alginate microparticles incorporating Aeromonas hydrophila designed for fish oral vaccination. Process Biochemistry. 2006;41(3):638-43.

28.	Romalde JL, Luzardo-Alvárez A, Ravelo C, Toranzo AE, Blanco-Méndez J. Oral immunization using alginate microparticles as a useful strategy for booster vaccination against fish lactoccocosis. Aquaculture. 2004;236(1):119-29.

29.	van der Lubben IM, Verhoef JC, van Aelst AC, Borchard G, Junginger HE. Chitosan microparticles for oral vaccination: preparation, characterization and preliminary in vivo uptake studies in murine Peyer's patches. Biomaterials. 2001;22(7):687-94.

30.	Azevedo AF, Galhardas J, Cunha A, Cruz P, Gonçalves LM, Almeida AJ. Microencapsulation of Streptococcus equi (italic) antigens in biodegradable microspheres and preliminary immunisation studies. Eur J Pharm Biopharm. 2006;64(2):131-7.

31.	Dubey S, Avadhani K, Mutalik S, Sivadasan SM, Maiti B, Paul J, et al. Aeromonas hydrophila (italic) OmpW PLGA Nanoparticle Oral Vaccine Shows a Dose-Dependent Protective Immunity in rohu (Labeo rohita) (italic). Vaccines. 2016;4(2).

32.	Awad A, Madla CM, McCoubrey LE, Ferraro F, Gavins FKH, Buanz A, et al. Clinical translation of advanced colonic drug delivery technologies. Advanced drug delivery reviews. 2022;181:114076-.

33.	Embregts CWE, Forlenza M. Oral vaccination of fish: Lessons from humans and veterinary species. Developmental & Comparative Immunology. 2016;64:118-37.

34.	Halimi M, Alishahi M, Abbaspour MR, Ghorbanpoor M, Tabandeh MR. Efficacy of a Eudragit L30D-55 encapsulated oral vaccine containing inactivated bacteria (Lactococcus garvieae/Streptococcus iniae) (italic) in rainbow trout (Oncorhynchus mykiss) (italic). Fish & Shellfish Immunology. 2018;81:430-7.

35.	Hayat M, Mohd Yusoff MS, Samad MJ, Abdul Razak IS, Md Yasin IS, Thompson KD, et al. Efficacy of Feed-Based Formalin-Killed Vaccine of Streptococcus iniae (italic) Stimulates the Gut-Associated Lymphoid Tissues and Immune Response of Red Hybrid Tilapia. Vaccines. 2021;9(1).

36.	Singh S, Sl H. Eudragit and its Pharmaceutical Significance. <http://wwwpharmainfonet/satishsinghkadian/publications/eudragit-and-its-pharmaceutical-significance>. 2009.

37.	Inactive ingredient search for approved drug products [Internet]. 2021. Available from: <https://www.accessdata.fda.gov/scripts/cder/iig/index.Cfm>.

38.	Piganelli JD, Zhang JA, Christensen JM, Kaattari SL. Enteric coated microspheres as an oral method for antigen delivery to salmonids. Fish & Shellfish Immunology. 1994;4(3):179-88.

39.	LILLEHAUG A. Oral immunization of rainbow trout, Salmo gairdneri Richardson, against vibriosis with vaccines protected against digestive degradation. Journal of Fish Diseases. 1989;12(6):579-84.

40.	Silva AL, Soema PC, Slutter B, Ossendorp F, Jiskoot W. PLGA particulate delivery systems for subunit vaccines: Linking particle properties to immunogenicity. Human Vaccines & Immunotherapeutics. 2016;12(4):1056-69.

41.	Ismail MS, Siti-Zahrah A, Syafiq MRM, Amal MNA, Firdaus-Nawi M, Zamri-Saad M. Feed-based vaccination regime against streptococcosis in red tilapia, Oreochromis niloticus × Oreochromis mossambicus (italic). BMC Veterinary Research. 2016;12(1):194.

42.	Yun S, Jun JW, Giri SS, Kim HJ, Chi C, Kim SG, et al. Efficacy of PLGA microparticle-encapsulated formalin-killed Aeromonas hydrophila (italic) cells as a single-shot vaccine against A. hydrophila (italic) infection. Vaccine. 2017;35(32):3959-65.

43.	Bosworth BG, Small BC, Gregory D, Kim J, Black S, Jerrett A. Effects of rested-harvest using the anesthetic AQUI-S™ on channel catfish, Ictalurus punctatus (italic), physiology and fillet quality. Aquaculture. 2007;262(2):302-18.

44.	Shoemaker C, Vandenberg G, Désormeaux A, Klesius P, Evans J. Efficacy of a Oralject (TM) Streptococcus iniae (italic) modified bacterin delivered using technology in Nile tilapia (Oreochromis niloticus) (italic). Aquaculture. 2006;255:151-6.

45.	Services SS. Comparing Two Proportions – Sample Size 2022 [Available from: <https://select-statistics.co.uk/calculators/sample-size-calculator-two-proportions/>.

46.	Animals (Scientific Procedures) Act 1986, (2012).

47.	Amend DF. Potency testing of fish vaccines. International Symposium on Fish Biologics: Serodiagnostics and Vaccines: Developments in Biological Standardisation; 1981. p. 447-54.

48.	Hogt AH, Dankert J, Feijen J. Adhesion of coagulase-negative staphylococci to methacrylate polymers and copolymers. J Biomed Mater Res. 1986;20(4):533-45.

49.	An YH, Friedman RJ. Concise review of mechanisms of bacterial adhesion to biomaterial surfaces. Journal of Biomedical Materials Research. 1998;43(3):338-48.

50.	Rosca ID, Watari F, Uo M. Microparticle formation and its mechanism in single and double emulsion solvent evaporation. Journal of Controlled Release. 2004;99(2):271-80.

51.	Payne AI. Gut ph and digestive strategies in estuarine grey mullet (Mugilidae) and tilapia (Cichlidae). Journal of Fish Biology. 1978;13(5):627-9.

52.	Heng HG, Ong TW, Hassan MD. Radiographic assessment of gastric emptying and gastrointestinal transit time in hybrid tilapia (Oreochromis niloticus × O. mossambicus) (italic). Vet Radiol Ultrasound. 2007;48(2):132-4.

53.	de Oliveira CG, López-Olmeda JF, Costa LS, do Espirito Santo AH, dos Santos FAC, Luz RK, et al. Gastrointestinal emptying and daily patterns of activity of proteinolytic enzymes in Nile tilapia (Oreochromis niloticus) (italic). Aquaculture. 2022;546:737338.

54.	L Zhang WY, C Hu, Q Wang, Y Wu. Properties and applications of nanoparticle/microparticle conveyors with adjuvant characterisitics suitable for oral vaccination. International Journal of Nanomedicine. 2018;13:2973-87.

55.	Smith NC, Rise ML, Christian SL. A Comparison of the Innate and Adaptive Immune Systems in Cartilaginous Fish, Ray-Finned Fish, and Lobe-Finned Fish. Frontiers in Immunology. 2019;10.

56.	Rombout JH, Abelli L, Picchietti S, Scapigliati G, Kiron V. Teleost intestinal immunology. Fish Shellfish Immunol. 2011;31(5):616-26.

57.	Delannoy CMJ, Crumlish M, Fontaine MC, Pollock J, Foster G, Dagleish MP, et al. Human Streptococcus agalactiae strains in aquatic mammals and fish. BMC Microbiology. 2013;13(1):41.

58.	Leal CAG, Queiroz GA, Pereira FL, Tavares GC, Figueiredo HCP. Streptococcus agalactiae Sequence Type 283 in Farmed Fish, Brazil. Emerg Infect Dis. 2019;25(4):776-9.
