# Supplementary material for: An oral pH-responsive Streptococcus agalactiae vaccine formulation provides protective immunity to pathogen challenge in tilapia: A proof-of-concept study
Source: PLoS One. 2023 Mar 3;18(3):e0278277. doi: 10.1371/journal.pone.0278277 (PMC9983853; doi:10.1371/journal.pone.0278277)
Supplement: Attachment — Submitted filename: Report.docx [file pone.0278277.s002.docx]

**Dear Editor**

Greeting,

The manuscript under title ( An oral pH-responsive Streptococcus agalactiaevaccine formulation provides protective immunity to pathogen challenge in tilapia: a proof-of-concept study ).

This study provides good findings that are helpful in minimizing the use of

antimicrobials and antibiotic resistance in aquaculture using oral vaccine . However, I found some points should be clarified.

**Constructive Comments to the author**

-The language of the text needs more revisions.

**Specific comments**

**Abstract**

    The abstract section should be briefly introduce the research background and research significance and clarify the research methods, then introduce the main research results, and finally, give the corresponding conclusions. I hope to make a comprehensive modification.

**Line 27-30**	Add this paragraph to the introduction

**Introduction**

The novelty should be highlighted more in the introduction. Additionally , organize it to be simple.

**Materials and methods**

Organize the materials to be simple in writing**.**

**Line 169**	Improve this title

**Line 176**	Put title for this paragraph

**Line 191**	what do you mean 0.7ml at 10^6^ cfu/mL ? especially at . Did you mean from? How do you adjust it?

**Line 244** you wrote, Red tilapia (*Oreochromis* sp.) was purchased. What is the name of the place ?

**Results**

The authors can improve the results by rewriting some sentences. Please, highlight just the main results to keep it short.

**Discussion**

The discussion should concatenate the results of the entire experiment into a reasonable story, but the discussion here is too scattered and not focused.

Add updated references for the paragraphs

**Line 439 - 453** Add references to this paragraph. However, it is a long paragraph .
