# Supplementary material for: An oral pH-responsive Streptococcus agalactiae vaccine formulation provides protective immunity to pathogen challenge in tilapia: A proof-of-concept study
Source: PLoS One. 2023 Mar 3;18(3):e0278277. doi: 10.1371/journal.pone.0278277 (PMC9983853; doi:10.1371/journal.pone.0278277)
Supplement: Attachment — Submitted filename: Response to Reviewers.docx [file pone.0278277.s003.docx]

We thank the reviewers for their constructive comments, and we have revised the manuscript extensively. Changes are shown in red font where possible. Much text has been removed so, this change cannot be shown. Also, to reduce the amount of red font, only the beginning of certain paragraphs that were changed are shown in red.

When submitting your revision, we need you to address these additional requirements.

1. Please ensure that your manuscript meets PLOS ONE's style requirements, including those for file naming. The PLOS ONE style templates can be found at 
[https://journals.plos.org/plosone/s/file?id=wjVg/PLOSOne_formatting_sample_main_body.pdf](https://eur01.safelinks.protection.outlook.com/?url=https%3A%2F%2Fprotect-au.mimecast.com%2Fs%2FxYpSCYW8Noc3XDKKpfZ2rAq%3Fdomain%3Djournals.plos.org&data=05%7C01%7Cs.murdan%40ucl.ac.uk%7C0586f686575e413691cb08daaf0efe1a%7C1faf88fea9984c5b93c9210a11d9a5c2%7C0%7C0%7C638014778479856896%7CUnknown%7CTWFpbGZsb3d8eyJWIjoiMC4wLjAwMDAiLCJQIjoiV2luMzIiLCJBTiI6Ik1haWwiLCJXVCI6Mn0%3D%7C3000%7C%7C%7C&sdata=sRtCK0JmSCg2fmXNlWRsJD4ZRZzY3F7my50VScgjpn8%3D&reserved=0) and 
[https://journals.plos.org/plosone/s/file?id=ba62/PLOSOne_formatting_sample_title_authors_affiliations.pdf](https://eur01.safelinks.protection.outlook.com/?url=https%3A%2F%2Fprotect-au.mimecast.com%2Fs%2F57ujCZY1NqiMY7118iXeAPT%3Fdomain%3Djournals.plos.org&data=05%7C01%7Cs.murdan%40ucl.ac.uk%7C0586f686575e413691cb08daaf0efe1a%7C1faf88fea9984c5b93c9210a11d9a5c2%7C0%7C0%7C638014778479856896%7CUnknown%7CTWFpbGZsb3d8eyJWIjoiMC4wLjAwMDAiLCJQIjoiV2luMzIiLCJBTiI6Ik1haWwiLCJXVCI6Mn0%3D%7C3000%7C%7C%7C&sdata=R3HSTZ5FScMDvR3mI%2FccmZ9JUWke5%2FD8BYOecV0mxqY%3D&reserved=0)

We have addressed all PLOS ONE's style requirements, apart from one. We have used Vancouver for the reference style as per PLOS ONE requirements. However, as we are using Endnote Web, the citations are coming as (X), rather than [X] i.e. square brackets. Endnote Web does not have the capability to change () to []. We could use ‘Numbered’ endnote style, but then we don’t get the first 6 authors listed in the bibliography. So we had to keep to Vancouver style. Would it be possible for the desk editors to convert the () into []? We did not do this ourselves as this would entail converting citations to plain text, in which case, we would lose the reference links.

2. Thank you for stating the following financial disclosure: 
 "SM received an internal UCL award funded through Research England’s ‘QR Global Challenges Research Fund’ ([https://www.ucl.ac.uk/](https://eur01.safelinks.protection.outlook.com/?url=https%3A%2F%2Fprotect-au.mimecast.com%2Fs%2FFubKC1WLPxcpX6rrBFOav8e%3Fdomain%3Ducl.ac.uk%2F&data=05%7C01%7Cs.murdan%40ucl.ac.uk%7C0586f686575e413691cb08daaf0efe1a%7C1faf88fea9984c5b93c9210a11d9a5c2%7C0%7C0%7C638014778480013129%7CUnknown%7CTWFpbGZsb3d8eyJWIjoiMC4wLjAwMDAiLCJQIjoiV2luMzIiLCJBTiI6Ik1haWwiLCJXVCI6Mn0%3D%7C3000%7C%7C%7C&sdata=%2FHwczLQX6oGqLBMOgyeDp9sjZ66LOXwjm8rHFdlliDI%3D&reserved=0)) for staff (SB) and experiments conducted at UCL and at Hue University.  Furthermore,  UCL,  Hue University ([https://hueuni.edu.vn/portal/en/](https://eur01.safelinks.protection.outlook.com/?url=https%3A%2F%2Fprotect-au.mimecast.com%2Fs%2FzWKxC2xMQzikQVLLZfL8aRJ%3Fdomain%3Dhueuni.edu.vn%2F&data=05%7C01%7Cs.murdan%40ucl.ac.uk%7C0586f686575e413691cb08daaf0efe1a%7C1faf88fea9984c5b93c9210a11d9a5c2%7C0%7C0%7C638014778480013129%7CUnknown%7CTWFpbGZsb3d8eyJWIjoiMC4wLjAwMDAiLCJQIjoiV2luMzIiLCJBTiI6Ik1haWwiLCJXVCI6Mn0%3D%7C3000%7C%7C%7C&sdata=cVwSkDa3fdzUqR28YLJWMF4uJfhPk02gf4YlBNuOc2k%3D&reserved=0)), The University of Sydney ([https://www.sydney.edu.au/](https://eur01.safelinks.protection.outlook.com/?url=https%3A%2F%2Fprotect-au.mimecast.com%2Fs%2FVFuaC3QNPBimvXjj9HpStI6%3Fdomain%3Dsydney.edu.au%2F&data=05%7C01%7Cs.murdan%40ucl.ac.uk%7C0586f686575e413691cb08daaf0efe1a%7C1faf88fea9984c5b93c9210a11d9a5c2%7C0%7C0%7C638014778480013129%7CUnknown%7CTWFpbGZsb3d8eyJWIjoiMC4wLjAwMDAiLCJQIjoiV2luMzIiLCJBTiI6Ik1haWwiLCJXVCI6Mn0%3D%7C3000%7C%7C%7C&sdata=LzOEnlXMRlT0yf7gBdjio0AKB495AXBTTwsqVa%2Fc070%3D&reserved=0)) and Harper Adams University ([https://www.harper-adams.ac.uk/](https://eur01.safelinks.protection.outlook.com/?url=https%3A%2F%2Fprotect-au.mimecast.com%2Fs%2FUAkFC4QOPEiJxzZZ9f5FsdD%3Fdomain%3Dharper-adams.ac.uk%2F&data=05%7C01%7Cs.murdan%40ucl.ac.uk%7C0586f686575e413691cb08daaf0efe1a%7C1faf88fea9984c5b93c9210a11d9a5c2%7C0%7C0%7C638014778480013129%7CUnknown%7CTWFpbGZsb3d8eyJWIjoiMC4wLjAwMDAiLCJQIjoiV2luMzIiLCJBTiI6Ik1haWwiLCJXVCI6Mn0%3D%7C3000%7C%7C%7C&sdata=zKetRm7ZHMXYTPRnBzvaKPws7aR%2BcLt8%2BGRPNp5if2A%3D&reserved=0)) further funded SM’s, NNP’s, RZ’s and TH’s time on the project. There were no grant numbers. The funders had no role in study design, data collection and analysis, decision to publish, or preparation of the manuscript."

At this time, please address the following queries:

a) Please clarify the sources of funding (financial or material support) for your study. List the grants or organizations that supported your study, including funding received from your institution. 
b) State what role the funders took in the study. If the funders had no role in your study, please state: “The funders had no role in study design, data collection and analysis, decision to publish, or preparation of the manuscript.”
c) If any authors received a salary from any of your funders, please state which authors and which funders.
d) If you did not receive any funding for this study, please state: “The authors received no specific funding for this work.”

Please include your amended statements within your cover letter; we will change the online submission form on your behalf.

*This is now implemented in the cover letter.*

3. In your Data Availability statement, you have not specified where the minimal data set underlying the results described in your manuscript can be found. PLOS defines a study's minimal data set as the underlying data used to reach the conclusions drawn in the manuscript and any additional data required to replicate the reported study findings in their entirety. All PLOS journals require that the minimal data set be made fully available. For more information about our data policy, please see [http://journals.plos.org/plosone/s/data-availability](https://eur01.safelinks.protection.outlook.com/?url=https%3A%2F%2Fprotect-au.mimecast.com%2Fs%2FCl7xC5QPXJi01MyypsVtneU%3Fdomain%3Djournals.plos.org&data=05%7C01%7Cs.murdan%40ucl.ac.uk%7C0586f686575e413691cb08daaf0efe1a%7C1faf88fea9984c5b93c9210a11d9a5c2%7C0%7C0%7C638014778480013129%7CUnknown%7CTWFpbGZsb3d8eyJWIjoiMC4wLjAwMDAiLCJQIjoiV2luMzIiLCJBTiI6Ik1haWwiLCJXVCI6Mn0%3D%7C3000%7C%7C%7C&sdata=IC0tLE2paFxOZdSqVgwHZI0So8Eh1gsiC1VH4CfZrz0%3D&reserved=0).

"Upon re-submitting your revised manuscript, please upload your study’s minimal underlying data set as either Supporting Information files or to a stable, public repository and include the relevant URLs, DOIs, or accession numbers within your revised cover letter. For a list of acceptable repositories, please see [http://journals.plos.org/plosone/s/data-availability#loc-recommended-repositories](https://eur01.safelinks.protection.outlook.com/?url=https%3A%2F%2Fprotect-au.mimecast.com%2Fs%2FhYPuC6XQ4LfoJPRR0SQwKbT%3Fdomain%3Djournals.plos.org&data=05%7C01%7Cs.murdan%40ucl.ac.uk%7C0586f686575e413691cb08daaf0efe1a%7C1faf88fea9984c5b93c9210a11d9a5c2%7C0%7C0%7C638014778480013129%7CUnknown%7CTWFpbGZsb3d8eyJWIjoiMC4wLjAwMDAiLCJQIjoiV2luMzIiLCJBTiI6Ik1haWwiLCJXVCI6Mn0%3D%7C3000%7C%7C%7C&sdata=1C%2FgOjVWxA95TDXbfqliHPqUTLtswLhmUAeDfp5VAgg%3D&reserved=0). Any potentially identifying patient information must be fully anonymized.

Important: If there are ethical or legal restrictions to sharing your data publicly, please explain these restrictions in detail. Please see our guidelines for more information on what we consider unacceptable restrictions to publicly sharing data: [http://journals.plos.org/plosone/s/data-availability#loc-unacceptable-data-access-restrictions](https://eur01.safelinks.protection.outlook.com/?url=https%3A%2F%2Fprotect-au.mimecast.com%2Fs%2FM6ViC71R2NTmYzrrVH15k9y%3Fdomain%3Djournals.plos.org&data=05%7C01%7Cs.murdan%40ucl.ac.uk%7C0586f686575e413691cb08daaf0efe1a%7C1faf88fea9984c5b93c9210a11d9a5c2%7C0%7C0%7C638014778480013129%7CUnknown%7CTWFpbGZsb3d8eyJWIjoiMC4wLjAwMDAiLCJQIjoiV2luMzIiLCJBTiI6Ik1haWwiLCJXVCI6Mn0%3D%7C3000%7C%7C%7C&sdata=phZYEBPahhGFx4BkVyp61QGfCTWr7o6o0z9IPdYk01s%3D&reserved=0). Note that it is not acceptable for the authors to be the sole named individuals responsible for ensuring data access.

We will update your Data Availability statement to reflect the information you provide in your cover letter.

4. We note that you have stated that you will provide repository information for your data at acceptance. Should your manuscript be accepted for publication, we will hold it until you provide the relevant accession numbers or DOIs necessary to access your data. If you wish to make changes to your Data Availability statement, please describe these changes in your cover letter and we will update your Data Availability statement to reflect the information you provide.

*data is now available at*

*Murdan, Sudax; Phuoc, Nguyen Ngoc; Basit, Abdul; N. Zadoks, Ruth; Herath, Tharangani; Bashir, Shazia (2022): An oral pH-responsive Streptococcus agalactiae vaccine formulation provides protective immunity to pathogen challenge in tilapia. University College London. Dataset.* [*https://doi.org/10.5522/04/19369376.v1*](https://eur01.safelinks.protection.outlook.com/?url=https%3A%2F%2Fdoi.org%2F10.5522%2F04%2F19369376.v1&data=05%7C01%7Cs.murdan%40ucl.ac.uk%7Ca6534ad49f9e46d7834308dab72ebca3%7C1faf88fea9984c5b93c9210a11d9a5c2%7C0%7C0%7C638023708599697305%7CUnknown%7CTWFpbGZsb3d8eyJWIjoiMC4wLjAwMDAiLCJQIjoiV2luMzIiLCJBTiI6Ik1haWwiLCJXVCI6Mn0%3D%7C3000%7C%7C%7C&sdata=UBaBVj6N9NWRcm2sY3SB6mnZq0GuL5y8QMWRzmyesFA%3D&reserved=0)

*Also stated in the revised cover letter.*

5. Please include your full ethics statement in the ‘Methods’ section of your manuscript file. In your statement, please include the full name of the IRB or ethics committee who approved or waived your study, as well as whether or not you obtained informed written or verbal consent. If consent was waived for your study, please include this information in your statement as well.

*The name of the ethics committee who approved the study is added in the main body. Written consent was also obtained from University College London, regarding the conduct of experiments in fish.*

[Note: HTML markup is below. Please do not edit.]

Reviewers' comments:

Reviewer #1: This is an interesting study detailing a pH-responsive carrier with formalin-killed whole cell oral vaccine against streptococcus agalactiae ST283 infection in tilapia. However, there are several concerns listed below, for the authors to improve the manuscript.

Major concerns:
1. The Introduction and Discussion sections should be rewritten because authors confused these two parts. Introduction should be included the context, the scope and the importance of your research. In the Discussion chapter, you need summarized the key findings, did not repeat all the results and related the importance of these key findings with the existing scholarly works and so on.

*The introduction and discussion have been rewritten and refocused in accordance with the feedback from Reviewers 1 and 3.*

2. The authors should try to acknowledge the limitations of the study in the Discussion chapter, especially in antibody detection and immune response after vaccination.

*We have added to the discussion chapter that antibody profiles were not determined. It is not known whether vaccine-induced immunity is purely anti-body mediated, so we considered a protective immune response, as observed in our study, a more important read-out*.

3. All the figures resolution was not high.
*This is now addressed.*

Minor comments:
1. In line 50, Keywords should be “oral vaccine, tilapia, streptococcus agalactiae, microparticles”

*Implemented as suggested*

2. In line 251, author said “Mean body weight of the tilapia used in this study was 15 ± 2 g”, Why select this age for vaccination? Add references.

*This is addressed. Reference has been added.*

3. In line 517-8, “further studies must be conducted to evaluate the efficacy of the encapsulated GBS when incorporated in fish feed and compare to unencapsulated GBS”. Capsular is widely accepted as important protective antigen of Streptococci, so this sentence should be deleted.

*There appears to be confusion between encapsulation of a vaccine to make it pH responsive, and the capsular antigens on the surface of the bacteria. We have rephrased the text to avoid such confusion. Interestingly, capsule is an important virulence factor in human GBS but not in animal GBS. We note this here for the reviewer’s interest but have not added this to the manuscript because that addresses a different type of encapsulation. We have used the word ‘entrapment’ to avoid any confusion.*

Reviewer #2: Authors should revise the manuscript as in the attached word file.

*Thank you for your detailed feedback on our manuscript. To accommodate the remaining reviewers’ suggestions, we rewrote some sections of the text. Where still relevant, we have incorporated your corrections.*

Line 178: Authors should add reference or NCBI GenBank accession number.

*An NCBI Genbank accession number was not provided because this isolate has not been sequenced. Use of field isolates to develop challenge models is customary in peer-reviewed tilapia research.*

Please revise italics in the reference section

*Implemented as suggested.*

All figures should be of high resolution

*This is now addressed.*

Reviewer #3: Dear Editor

Greeting,
The manuscript under title ( An oral pH-responsive Streptococcus agalactiaevaccine formulation provides protective immunity to pathogen challenge in tilapia: a proof-of-concept study ).
This study provides good findings that are helpful in minimizing the use of
antimicrobials and antibiotic resistance in aquaculture using oral vaccine . However, I found some points should be clarified.
*Thank you for your positive feedback and your suggestion to improve accessibility of the information.*

Constructive Comments to the author
-The language of the text needs more revisions.
*Revisions have been made to all sections of the text, including Abstract, Introduction, Methods, Results and Discussion.*

Specific comments

Abstract

The abstract section should be briefly introduce the research background and research significance and clarify the research methods, then introduce the main research results, and finally, give the corresponding conclusions. I hope to make a comprehensive modification.
*The abstract has been rewritten using the suggested structure.*

Line 27-30 Add this paragraph to the introduction

*This information is covered in the introduction.*

Introduction

The novelty should be highlighted more in the introduction. Additionally , organize it to be simple.
*We have simplified the introduction so that it takes the reader through the importance of intensification of aquaculture for food security, and the resultant risk of disease emergence, to the need for disease control measures, which include antimicrobials and vaccines. Both have disadvantages and limitations, which are summarized briefly, leading logically to the need for better options, e.g., targeted oral vaccination. The current state of knowledge around this option is introduced in such a way that the novelty of our approach is highlighted more clearly. The introduction ends with a shortened and simplified description of the study’s aim, design, and key findings.*

Materials and methods
Organize the materials to be simple in writing.

*We have simplified the organization and the writing and checked with non-native speakers whether they found the text easy to understand.*

Line 169 Improve this title

*This title has been improved*

Line 176 Put title for this paragraph

*The improved title for the previous paragraph now also covers this paragraph.*

Line 191 what do you mean 0.7ml at 106 cfu/mL ? especially at . Did you mean from? How do you adjust it?

*No, we mean at a concentration of 10^6^ CFU/mL.*

*0.7 mL was written in the text as 0.7 ml of this GBS suspension was placed in a Cuvette, which was then placed in the instrument, and measurements were then taken. We have removed 0.7ml from the text now to avoid any confusion and as it is not needed. Concentration was adjusted by dilution, prior to placement of the suspension in the cuvette.*

Line 244 you wrote, Red tilapia (Oreochromis sp.) was purchased. What is the name of the place ?
*Name of the place, Provincial Breeding Centre of Thua Thien Hue at Cu Chanh Commune, Thua Thien Hue province, Vietnam, is in the text.*

Results
The authors can improve the results by rewriting some sentences. Please, highlight just the main results to keep it short.

*The results section has been shortened, and several sentences have been rewritten.*

Discussion
The discussion should concatenate the results of the entire experiment into a reasonable story, but the discussion here is too scattered and not focused.

*Discussion has been rewritten and refocused in accordance with the reviewers’ feedback.*

Add updated references for the paragraphs
Line 439 - 453 Add references to this paragraph. However, it is a long paragraph.

*This paragraph has been removed when the manuscript has been simplified and refocused.*
